# Recent advances in the understanding, detection and therapeutic targeting of bacterial recalcitrance

**DOI:** 10.1186/s12866-025-04210-1

**Published:** 2025-08-08

**Authors:** Elsa Roch, Jérémie Ducrocq, Nicolas Jacquier

**Affiliations:** 1 School of Biology, University of Lausanne, Lausanne, 1015 Switzerland; 2 Institute of Microbiology, University Hospital and University of Lausanne, Lausanne, 1011 Switzerland

**Keywords:** Bacterial tolerance, Bacterial persistence, Recalcitrance, Dormancy, Stringent response, SOS response, Biofilm, Antimicrobial agents, Antibiotics

## Abstract

Antibiotic resistance is a growing threat for modern medicine, making treatment of infectious diseases increasingly tedious. However, even non-resistant bacteria can survive treatment and cause recurrent infections. This phenomenon is often due to non-proliferating bacteria able to survive the treatment and to resume infection afterwards, also called recalcitrant bacteria. Bacterial recalcitrance, which encompasses tolerance and persistence, is defined by increased survival of bacteria in the presence of antimicrobial agents. In contrast to resistance, the mechanisms underlying recalcitrance are only partially understood.

In this review, we summarise the recent advances in the understanding of recalcitrance, its detection, as well as anti-recalcitrance therapies that have been developed. Recalcitrance is thought to be caused by a reduction of bacterial metabolism, mostly driven by stringent and SOS responses, leading to bacterial dormancy. These dormant bacteria escape the action of many antibiotics, preventing the complete resolution of infection. However, strategies have been proposed to tackle recalcitrance. Recalcitrant bacteria are susceptible to drugs whose action is independent of metabolic activity, such as membrane-targeting compounds. Inhibitors blocking the entry of bacteria into dormancy or locking bacteria in a permanent state of dormancy could help avoid recurrence of the infection. Dormant bacteria could also be forced to resume growth through supply of nutrients or signalling molecules. A phage specifically targeting dormant bacteria was recently described and may be an important tool to fight bacterial recalcitrance.

Recalcitrance has been neglected for a long time, being in the shadow of resistance. However, both phenomena need to be further investigated in the future to develop a complete array of antibacterial agents that will allow to permanently eradicate all types of bacterial infections.

## Background

In the beginning of the twentieth century, bacterial infections, such as pneumonia or tuberculosis were important causes of death. In the 1940s, the discovery of penicillin by Alexander Flemming started the Golden age of antibiotics. However, not long after these antimicrobial compounds started to be used on a large scale, resistance appeared. Antibiotic resistance has developed to such an extent that nowadays the alarm bells are ringing to combat it. Indeed, antibiotic treatment failures caused by multidrug resistant bacteria may set medicine back to a stage similar to the pre-antibiotic era [1].

Besides resistance, persistence and tolerance (grouped together under the term recalcitrance, see “Definitions”) are also a major cause of antibiotic treatment failure, which has been largely underestimated [2, 3]. Compared to bacterial resistance, recalcitrance has been understudied and is still not very well understood, despite characterization of persistence and tolerance dating back to the 1940s [4, 5]. However, persistence reawakened the interest of the scientific community only in the 1980s through the discovery of a highly persistent mutant of *Escherichia coli* [6]. In 2000, Lewis et al. discovered a link between persistence and biofilms in *Pseudomonas aeruginosa* [7]. As bacterial biofilms are often involved in chronic infections, this discovery highlighted an interest in studying persistent and tolerant bacteria in the context of chronic infections [8]. The number of publications addressing persistence and tolerance greatly increased during the past 10 years, however definitions of persistence and tolerance found in these publications can vary and not always follow guidelines proposed by a consortium of specialists in the field [2].

Recalcitrance was observed in a wide variety of pathogens, such as *Mycobacterium tuberculosis, Yersinia pseudotuberculosis, Salmonella enterica* and *Staphylococcus aureus* [9]. *S. aureus*, for example, can cause chronic and recurrent bacteraemia, which are tedious to treat and induce high rates of morbidity and mortality. Importantly, *S. aureus* has the capacity to survive and proliferate intracellularly, which makes the access to bacteria more difficult for antibiotics. This intracellular localisation combined with recalcitrance can lead to antibiotic treatments failure [10].

In this review, we summarise current understanding of tolerance and persistence mechanisms, concentrating on recent advances in the characterization of these phenomena. In a second part we discuss recent advances made in the detection and diagnosis of recalcitrant infections. Finally, we describe potential strategies currently investigated to therapeutically target recalcitrant cells.

## Definitions

These definitions mostly follow the guidelines that were proposed during the workshop “Bacterial Persistence and Antimicrobial Therapy” organized by the European Molecular Biology Organization (EMBO) in 2018 [2].

### Resistance

Resistance is the capacity of bacteria to proliferate in the presence of a normally inhibitory concentration of an antibiotic. It is characterized by a higher minimum inhibitory concentration (MIC) of the antibiotic. Resistance is caused by genetic modifications that can be acquired by spontaneous mutations or horizontal gene transfer.

### Tolerance

Tolerance is the ability of bacterial population to exhibit prolonged survival to antibiotic treatment. Tolerance is due to homogeneous phenotypic changes in a bacterial population, enabling survival in the presence of an antibiotic. It is characterized by an unchanging MIC, but an augmentation of the minimum duration for killing 99% of the population (MDK99). A tolerant bacterial population is non-growing in presence of the antibiotic but can resume proliferation once the antibiotic is removed. Tolerance can be caused by changes in the bacterial environment (e.g. nutrient levels) and the level of tolerance development can be influenced by genetic mutations.

### Persistence

Persistence is a heterogeneous phenotypic change in a bacterial population, enabling a bacterial subpopulation to survive to a higher bactericidal antibiotic concentration (higher MDK99), without any change in MIC. Only a sub-part of the population, generally less than 0.1%, dies more slowly in presence of the antibiotic. This is characterized by a biphasic killing curve: in a first phase the susceptible population quickly dies and then the persistent subpopulation dies more slowly.

### Recalcitrance

The recalcitrance is a term, which groups tolerance and persistence, as recently proposed by Helaine et al. [11]. This term is used to improve brevity and clarity regarding concepts or characteristics that can be applied to both tolerance and persistence.

### Dormancy

Dormancy is a non-growing state of bacteria characterised by a highly reduced metabolic activity. It can cause tolerance and persistence. The level of dormancy, also called dormancy depth, can vary, depending on which bacterial metabolic activities are stopped, and at which level [12].

## Main text

### Tolerance and persistence mechanisms

**Fig. 1:**
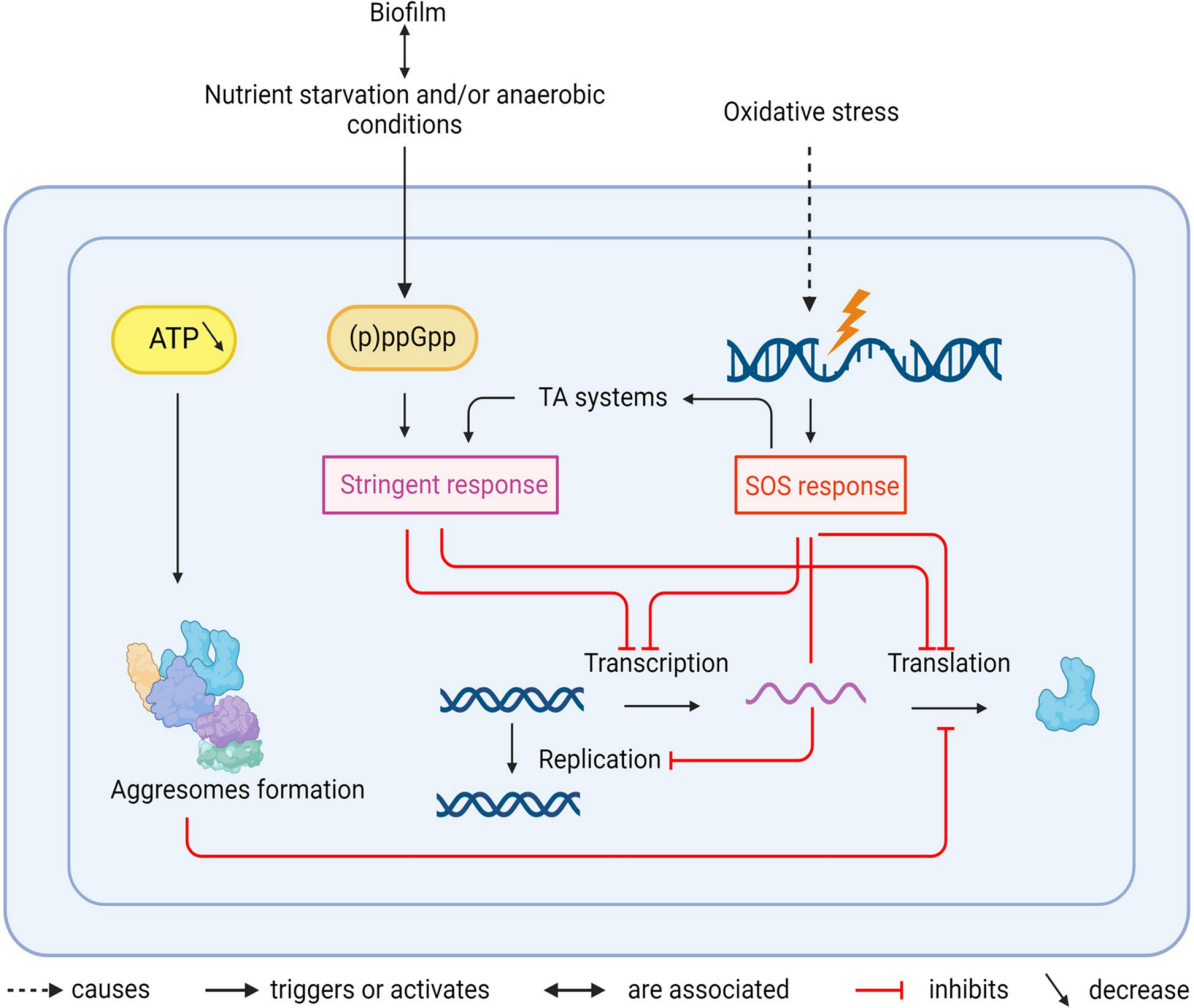
Schematic summary of mechanisms leading to tolerance and persistence in a bacterial cell. Black arrows indicate activations, red arrows inhibitions. TA: Toxin/Antitoxin

Recalcitrance is often linked to profound changes in cellular metabolism, which are listed below and in Fig. 1. Such changes can affect bacterial proliferation and make bacteria dormant. Dormancy is thus tightly linked to recalcitrance.

#### Dormancy is induced by profound changes in cellular metabolism

Various pathways can lead to dormancy, all including a drop in cellular metabolism, such as a decrease in levels of ATP production, replication, transcription, or translation [13]. However, level of dormancy can differ within a bacterial population. Depending on the dormancy depth, time required for bacteria to resume growth can vary. When a bacterial population encounters favourable conditions for proliferation, some bacteria quickly regrow, while others take hours to days to resuscitate [12].

Dormancy is partly induced by depletion of intracellular ATP, which subsequently causes the formation of aggregates of endogenous proteins called aggresomes [12]. Proteins, mainly involved in replication, transcription and translation, aggregate and cannot function normally, blocking essential processes and inducing dormancy. The disintegration of aggresomes is a critical step for dormant bacteria to resume growth (a process also called resuscitation). Aggresomes disintegration is mediated by ATP replenishment, through the activity of proteases and chaperones. In *E. coli,* DnaK and ClpB proteins are recruited for resuscitation. DnaK binds to aggresomes, allowing the recruitment of the disaggregase ClpB [12].

A recent study reveals that tolerant and persistent cells enter different levels of dormancy [14]. Tolerance implies that a large majority of a bacterial population enters dormancy and thus survives to antibiotic exposure. These bacteria enter deep dormancy, stopping the translation of protein for transcription and repair of DNA. In contrast, persistent bacteria slow down their metabolism by reducing the production of ribosomal proteins and proteins involved in carbon metabolism and oxidative phosphorylation but keep a low level of transcription and translation [14]*.*

#### (p)ppGpp and the stringent response play a major role in bacterial recalcitrance

(p)ppGpp (guanosine penta/tetraphosphate) is a small molecule, which plays a predominant role during nutrient starvation, activating the so-called stringent response. In Gram-negative bacteria, (p)ppGpp is produced especially during starvation by proteins of the RelA/SpoT family. In Gram-positive bacteria, (p)ppGpp is controlled by Rel, which functions as both (p)ppGpp synthetase and hydrolase [15]. (p)ppGpp inhibits on one hand transcription and translation to slow down the metabolism of the bacteria. It also activates the expression of stress response genes and acts as a coordinator of many stress response mechanisms [15]. (p)ppGpp can also modulate the quorum sensing activity. In *Pseudomonas aeruginosa*, (p)ppGpp is required for the expression of the two quorum sensing systems RHL and LAS [16]. (p)ppGpp may thus indirectly affect formation of biofilm, which is an important player in bacterial tolerance (See below). (p)ppGpp and the stringent response have thus a key function for bacterial recalcitrance [15, 17].

#### Toxin-Antitoxin systems are responsible for persistence penetration in bacterial population

Toxin-Antitoxin systems (TA systems) are composed of a toxin protein, which blocks a specific metabolic process, and of an antitoxin protein or RNA, which counteracts the toxin. Six different types of antitoxins have been described up to date, depending on the way they act to block the toxin (Reviewed in [18])*.* TA systems play an important role in persistence: an antimicrobial stress inactivates the antitoxins, allowing the toxins to block a metabolic process and therefore bacterial proliferation [8].

A well-known TA system is the HipAB system, which is widespread among Gram-negative species and was first described in *E. coli* [6]*.* The toxin HipA is a protein kinase that inhibits the glutamyl tRNA synthetase GltX by phosphorylating it. The inactivation of GltX leads to amino acid starvation and activation of the stringent response. The antitoxin HipB inactivates the toxin HipA directly by binding to it [19]. Upon a certain threshold of free HipA proteins, a subpopulation of cells enters dormancy and becomes persistent. The level of this threshold correlates with the level of penetration of persistence in the bacterial population. The heterogeneity in population is apparently caused by fluctuations of cellular quantity of toxin HipA, reaching or not the threshold. Mutations causing a decrease in the affinity of the toxin HipA for the antitoxin HipB, such as in the high-persistent mutant *hipA7*, can increase the percentage of population that enters dormancy [20].

### DNA damage caused by oxidative stress can lead to recalcitrance

#### ROS stress can induce recalcitrance both directly and indirectly

Reactive Oxygen Species (ROS) are produced by immune cells, macrophages in particular, in response to infection [21]. ROS such as superoxide (O^2−^) and hydroxyl radical (HO•) can damage DNA, lipids and proteins and cause the death of the bacterium [22]. However, these compounds can induce antibiotic tolerance, either through a direct activity on enzymes or indirectly via the SOS response [3]. They can directly inactivate aconitase, an enzyme of the Krebs cycle, as was shown in *S. aureus* infected macrophages [23]. In fact, ROS block the action of aconitase by attacking the Iron-Sulphur cluster in the active site of the enzyme. Consequently, the Krebs cycle is interrupted, bacteria synthesise low levels of ATP and their growth is slowed, inducing tolerance [23]. Beside ROS, nitric oxides can also induce recalcitrance through respiration and membrane potential inhibition [24, 25].

#### The SOS response is a mediator of dormancy

The SOS response is a bacterial process involved in the repair of damages to DNA made by ROS, among others [26]. SOS response causes a transient replication halt during which DNA can be repaired. It is regulated by two main actors: the repressor of the SOS regulon LexA and the sensor of DNA damage RecA. The repressor LexA is constitutively bound upstream of genes of the SOS regulon through a DNA sequence called the SOS box, thus repressing the expression of these genes when no DNA damages are detected. DNA damages induce accumulation of single stranded DNA that are recognized and bound by RecA. The formed nucleoprotein complex triggers the cleavage of LexA by autoproteolysis. SOS genes are thus expressed, including the *sulA* gene in *E. coli,* coding for a protein that stops cell division by sequestering the components of the cell division apparatus [27]. Furthermore, the SOS system includes a toxin-antitoxin system. IstR1, the antitoxin, is expressed at a basal level, whereas the toxin TisB is under LexA control [27]. When TisB concentration exceeds that of the antitoxin IstR1, free TisB forms pores in the bacterial inner membrane, decreasing the proton motive force and subsequently the activity of the ATP synthase [28]. Less ATP is produced, leading to dormancy of the bacterial cell**.**

Recently single bacteria RNAseq and CRISPR interference were used to identify common features of persister cells. This study suggests that Lon protease may be critical for persistence [29]. Lon is required to inactivate SulA to resume growth of recalcitrant bacteria. Deletion of either *lon* or *sulA* strongly decreases antibiotic survival [29].

### A potential role for pili in tolerance

Bacterial aggregation and changes in motility can modulate antibiotic recalcitrance [30, 31]. Key factors of bacterial aggregation and motility are type IV pili [32]. Modifications of type IV pili have been recently proposed to affect tolerance in *Neisseria gonorrhoeae* and *P. aeruginosa* [31, 33]. Type IV pili are filamentous appendages composed of pilin proteins and involved in adhesion and motility, among others. PilE of *N. gonorrhoeae* undergoes antigenic variation, allowing immune escape. However, this has consequences on adhesion properties, aggregation and motility. It was observed that *N. gonorrhoeae* strains with stronger aggregation capabilities were also more tolerant to antimicrobial agents [33].

In contrast, non-motile *P. aeruginosa* strains lacking type IV PilD protein show increased antibiotic persistence to antibiotics. Such strains are commonly involved in chronic infections [31]. Taken together, these recent results indicate that type IV pili may play different roles in recalcitrance in different bacterial species.

### Biofilm formation creates conditions conducive to persistence

Biofilms are the result of an aggregation of bacteria, generally through attachment to a substrate. These bacteria produce an extracellular matrix, composed of polysaccharides, proteins, extracellular DNA and other minor components [34]. This extracellular matrix acts as a very selective barrier, which makes the access to the bacteria more tricky for the antibiotics [35].

The persistence in biofilms can be the result of different mechanisms. In fact, biofilms create a microenvironment in which certain bacteria live in nutrient-poor, anaerobic conditions, especially at the core of the biofilm [35]. Bacteria in such an environment will have a low intracellular ATP level [36]. Consequently, starvation will trigger the expression of (p)ppGpp by the action of RelA/SpoT and therefore results in persistence [15].

### Detection and study of recalcitrant bacteria

Recalcitrance is not trivial to study, because of its transient and heterogenous nature. It has been recently shown that CFU measurement to assess survival of bacteria following an antimicrobial treatment may be misleading for some antibiotics such as fluoroquinolones, since plating intoxicated bacteria on rich media may cause their death through reactivation of their metabolism [37]. Complementary techniques are thus useful to strengthen our knowledge of recalcitrance. Indeed, striking progress has been made in the detection and study of recalcitrant bacteria. The development of prokaryotic single-cell RNA sequencing has provided an essential tool to study heterogeneity in a bacterial population [38]. This was used recently to identify genes involved in persistence [29]. Other techniques such as FACS [39], microfluidics [40], microscopy [41] and nanomotion [42] have been proposed to increase our knowledge of recalcitrance. Taken together, these techniques will hopefully allow an easier detection of recalcitrant bacteria for both research and diagnosis purposes.

### Persistence in vivo: a limited impact?

Persistence has been clearly demonstrated in vitro and its in vivo importance has been repeatedly pinpointed in literature (Reviewed in [43]). However, recent study by Fanous et al*.* investigated the effect of a selection of antibiotics towards a mouse model of *Salmonella* infection [37]. The limited efficacy of the antibiotics could not be explained by stress-induced persistence. In contrast, nutrient depletion was causing slow growth, and subsequently increased population-level tolerance to antibiotics [37]. This indicates that further research is required to fully apprehend the role of persistence in vivo.

### Strategies to eradicate recalcitrant bacteria

**Fig. 2:**
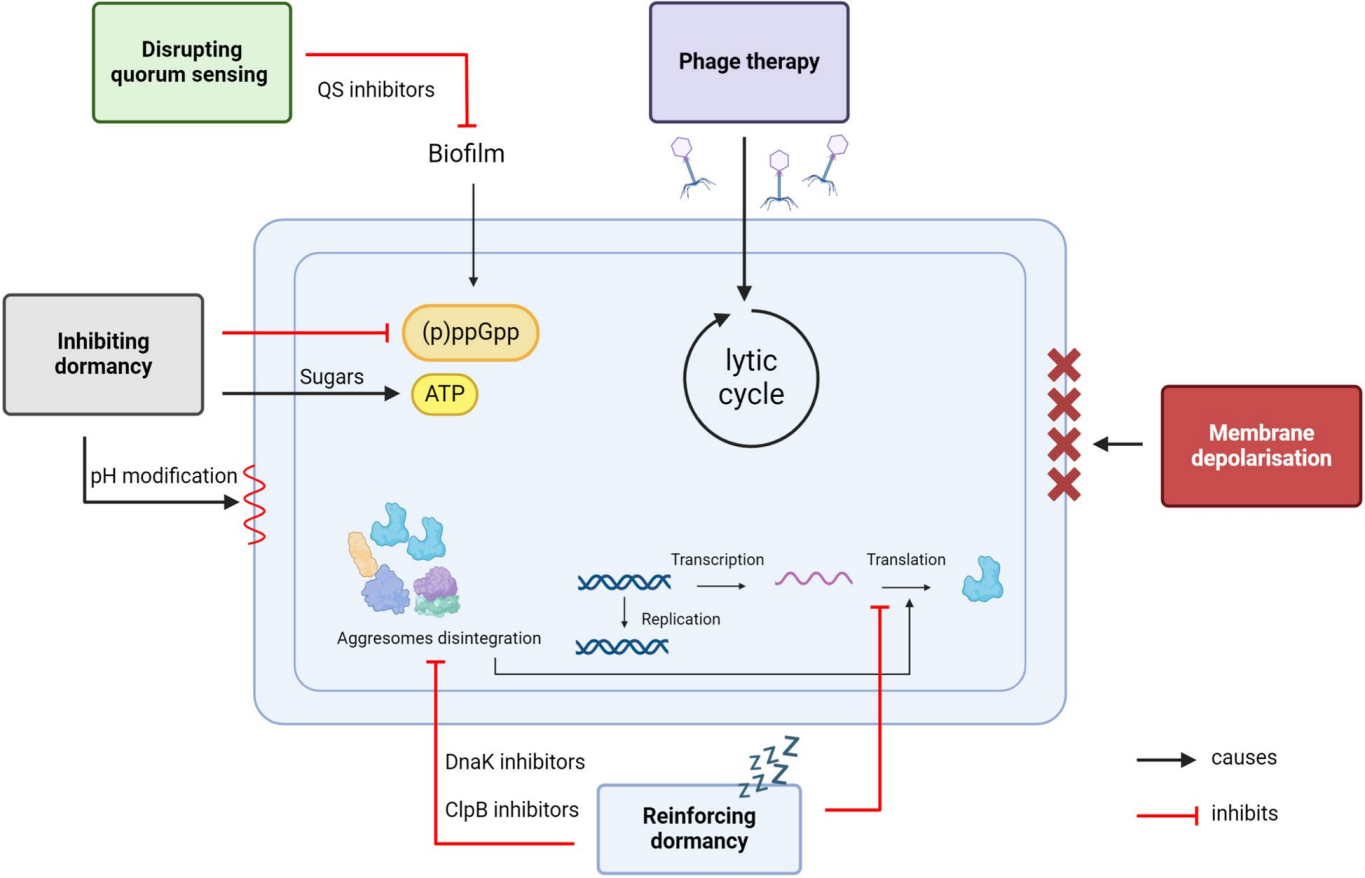
Schematic summary of potential treatment strategies for recalcitrant bacteria

Since recalcitrant bacteria have a low metabolic activity, most antibiotics targeting active processes of bacterial cells, such as inhibitors of DNA, RNA, proteins, cell wall or folate synthesis, are unable to eradicate them. There are thus ongoing attempts to develop strategies to specifically target these recalcitrant bacteria. These strategies consist mainly in (i) the development of antimicrobial agents that are efficient towards recalcitrant bacteria and (ii) the development of drugs that modulate recalcitrance, as detailed below and in Fig. 2.

#### Antimicrobial agents efficient towards recalcitrant bacteria

To be efficient towards recalcitrant bacteria, it is believed that antimicrobial agents should target processes that are independent of metabolic activity. Membrane active compounds are thus proposed as potential efficient anti-recalcitrant agents.

#### Membrane-active antimicrobial agents

Membrane disruptors directly target the structure, integrity, and function of the membrane [44]. Some antimicrobial peptides (AMPs) are membrane disruptors, making them potential anti-recalcitrance agents [45]. AMPs are short peptides, generally cationic, that are part of the innate immune defence of various organisms, including bacteria, plant, vertebrate and invertebrates [46]. Some AMPs that form pores in the membrane are effective on both metabolically active and dormant bacteria [47, 48]. This is for example the case for the AMP temporin G, which efficiently eradicates persistent *S. aureus* [47] and for LL-37 derived AMPs, which efficiently eradicate *B. subtilis* persisters, but not spores [39].

Polymyxins (Colistin and polymyxin B, among others) are polypeptidic antibiotics, used as reserve treatment towards infections caused by multi-resistant Gram-negative bacteria [49]. Polymyxins are positively charged polypeptides that bind to the outer membranes of Gram-negative bacteria via the negative charges of LPS [50]. They increase outer membrane permeability, pass into the periplasmic space and then disrupt the inner membrane, causing the bacteria to die. Colistin has been shown to be active against persistent *P. aeruginosa* in combination with other antibiotics [51].

#### Bacteriophages targeting dormant cells

Bacteriophages, as natural predators of bacteria, can efficiently eradicate persistent bacteria. Bacteriophage-based treatment could thus be used as a complement to antibiotics to kill recalcitrant bacteria. This was recently applied on *P. aeruginosa* biofilm [52]. In contrast, in some cases, persistent bacteria can escape phages activity [53]. It is thus essential to identify the proper phages able to eradicate recalcitrant bacteria. Recently, Paride, a *P. aeruginosa* phage was shown to be efficient towards dormant bacteria both in vitro and in vivo, through a lytic cycle [54]. This indicates that some phages are able to bypass the low metabolic state of dormant bacteria, by a mechanism that has still to be investigated, in order to make them produce a new generation of phages.

#### Development of modulators of recalcitrance

An alternative strategy to directly target recalcitrant bacteria is to modulate either entry in or exit from recalcitrance. By forcing bacteria out of dormancy, or inhibiting dormancy initiation, one can avoid bacteria to be recalcitrant to antibiotics. Alternately, bacteria could be forced into an irreversible state of dormancy, avoiding the proliferation of recalcitrant bacteria after removal of the antibiotic.

#### Awakening dormant cells to make them sensitive to antibiotics again

Awakening dormant cells could hit two birds with one stone: it may allow to ‘reset’ the metabolic processes targeted by the antibiotics, and to reactivate the energy-dependent influx pumps involved in antibiotic import. Metabolic stimulation with different kinds of sugars has been tested, with some success. Fumarate was identified as a potentiator of aminoglycoside towards tolerant *P. aeruginosa *in vitro [55]. This was also shown for tolerant *E. coli*, which could be sensitized to fluoroquinolones and other topoisomerase inhibitors through metabolic stimulation with several kinds of sugars [56]. Metabolization of sugars leads to ATP synthesis and reactivates proton motive force. A clinical trial (UROPOT) recently started, using mannitol as a metabolic stimulation in patients with asymptomatic *E. coli* and/or *Klebsiella pneumoniae* bacteriuria who undergo endourological surgeries [57]. This metabolic stimulation is aimed at eradicating bacteria tolerant to amikacin to avoid postsurgical complications.

Alternatively, antibiotic uptake can be activated through modification of pH gradient across bacterial membrane. Supplementation with L-arginine or L-glutamine, respectively, can modulate this pH gradient, thus enhancing aminoglycosides uptake [58, 59]. Effect of L-arginine in combination with tobramycin against *P. aeruginosa* biofilms was recently described, providing a potential treatment for recalcitrant *P. aeruginosa* in cystic fibrosis patients [60].

#### Inhibiting entry into dormancy

(p)ppGpp is an important signalling molecule of the stringent response, involved in regulation of dormancy [61]. RelA, the protein responsible for (p)ppGpp biosynthesis is thus a potential target to fight recalcitrance. However, only few inhibitors of (p)ppGpp biosynthesis have been identified. Relacin was the first described (p)ppGpp biosynthesis inhibitor active towards Gram-positive bacteria, but its efficiency and pharmacokinetics properties were not compatible with a clinical usage [62]. Several attempts have been performed to find other RelA inhibitors, with limited success [63–65]. More recently, tetrone lactones, molecules structurally similar to vitamin C were shown to inhibit (p)ppGpp biosynthesis. They efficiently inhibit recalcitrance of *Mycobacterium smegmatis*, a close relative of *M. tuberculosis* [66].

Inhibition of TA systems activation is another strategy explored to fight recalcitrance. In this context, the Lon protease was identified as a promising target, since absence of Lon strongly decreased survival to antibiotics treatment [29]. Lon inhibitors have thus been tested to inhibit recalcitrance [67]. Bortezomib is an example of Lon inhibitor, which may be used in the future as an anti-recalcitrance agent [68].

Inhibition of polyphosphate kinase was also highlighted as a potential strategy to fight recalcitrance. Inorganic polyphosphate is required for persistence of *A. baumannii*, among others [69]. It is produced by polyphosphate kinase (PPK), which was shown to be inhibited by scutellarein. Scutellarein, a naturally occurring flavonoid, lowers recalcitrance of *A. baumannii* both in vitro and in vivo [70].

By pursuing the investigation of recalcitrance mechanisms, novel drug targets will most probably be described in the future, opening new paths for the development of anti-recalcitrance compounds. It has however to be mentioned that dormancy inhibitors may have a limited clinical benefit, since they should be applied at the early onset of infection and could be inefficient on already installed chronic infections.

#### Making dormancy irreversible

Recent advances have been made in the understanding of mechanisms involved in dormancy. The description of aggresomes formation, along with the description of different levels of dormancy opens the door to alternative strategies aimed at locking bacteria in an irreversible state of dormancy. This could be performed by inhibiting the disaggregation of proteins involved in resuscitation. Inhibitors of DnaK or ClpB could be developed for this purpose as recently proposed by Pu and colleagues [12, 71].

#### Targeting the communication between bacteria to prevent biofilm formation

Quorum sensing is a major mediator of the communication between bacteria and plays a significant role in the biofilm formation. Therefore, it is a potential target for anti-persister treatments. Extensive ongoing research is performed in the field with numerous new potential quorum sensing inhibitors described recently, as reviewed elsewhere [72, 73]. However, quorum sensing inhibitors efficiency might be limited and have a rather narrow spectrum of action, since different bacterial species produce different quorum sensing molecules [74].

## Conclusions

While recalcitrance plays a significant role in antibiotic treatment failures, this phenomenon was for a long time in the shadow of antibiotic resistance. Even nowadays, the shade of meaning between tolerance, persistence and resistance is not clearly assessed in many publications. For this reason, we highlight in this review definitions that were based on the tremendous work of experts in the field who are trying to unify the nomenclature (2).

Mechanisms involved in bacterial recalcitrance are diverse, but generally involve stress response and low metabolic activity. Recalcitrant bacteria are difficult to detect and to identify. However, recent advances in the field may lead to a fast development of tools to investigate these bacteria. Given the importance of recalcitrance, a plethora of therapeutic strategies are currently under development. Using agents specifically targeting recalcitrant bacteria (phages, AMPs, among others) or by modulating dormancy entry or exit, we will hopefully tackle chronic and recurrent infections more efficiently.

## Data Availability

No datasets were generated or analysed during the current study.

## Abbreviations

AMPs: Antimicrobial peptides
LPS: Lipopolysaccharide
MDK99: Minimum duration for killing 99%
MIC: Minimal inhibitory concentration
(p)ppGpp: Guanosine penta/tetraphophate
PPK: Polyphosphate kinase
ROS: Reactive oxygen species
TA: Toxin/antitoxin
